# Single and dual antiplatelet therapy in elderly patients of medically managed myocardial infarction

**DOI:** 10.1186/s12877-018-0777-4

**Published:** 2018-04-05

**Authors:** Ting-Tse Lin, Hsiu-Yun Lai, K. Arnold Chan, Yen-Yun Yang, Chao-Lun Lai, Mei-Shu Lai

**Affiliations:** 1 Department of Internal Medicine, National Taiwan University Hospital Hsin-Chu Branch, Hsin-Chu, Taiwan; 2 Institute of Biomedical Engineering, National Chiao-Tung University, Hsin-Chu, Taiwan; 3 Department of Family Medicine, National Taiwan University Hospital Hsin-Chu Branch, Hsin-Chu, Taiwan; 4 Department of Medical Research, National Taiwan University Hospital, Taipei, Taiwan; 5 Graduate Institute of Oncology, College of Medicine, National Taiwan University, Taipei, Taiwan; 6 Center for Comparative Effectiveness Research, National Center of Excellence for Clinical Trial and Research, National Taiwan University Hospital, Taipei, Taiwan; 7 Center for critical care medicine, National Taiwan University Hospital Hsin-Chu Branch, Hsin-Chu, Taiwan; 8 Department of Internal Medicine, College of Medicine, National Taiwan University, Taipei, Taiwan; 9 Institute of Epidemiology and Preventive Medicine, College of Public Health, National Taiwan University, Taipei, Taiwan

**Keywords:** Aspirin, Clopidogrel, Dual/single antiplatelet therapy, Elderly AMI patients

## Abstract

**Backgrounds:**

To examine the comparative effectiveness between dual and single antiplatelet therapies in real-world, medically managed elderly patients with acute myocardial infarction (AMI).

**Methods:**

This retrospective study identified very elderly (> 85 years) patients, who were medically managed, with their first AMI from the Taiwan National Health Insurance claims database from 2007 to 2010. Patients were classified as dual antiplatelet therapy (DAPT) group, aspirin only group and clopidogrel only group. Study outcomes included all-cause death, cardiovascular death and gastrointestinal bleeding. Treating DAPT group as the reference, we employed a multivariable Cox regression model to compare the relative risks of outcomes between 3 groups using pairwise comparison approach.

**Results:**

Among 1469 patients with incident ST-elevation myocardial infarction (STEMI, 14%) or non-STEMI (86%), 390 patients were prescribed DAPT, 549 aspirin only, and 530 clopidogrel only. After 9 months of follow-up, aspirin only group had similar risks of all-cause death (adjusted HR 1.21, 95% CI 0.77–1.89, *p* = 0.41), cardiovascular death (adjusted HR 1.16, 95% CI 0.66–2.04, *p* = 0.60) and gastrointestinal bleeding (adjusted HR 1.66, 95% CI 0.77–3.57, *p* = 0.20) in comparison with DAPT group. Clopidogrel users had a higher risk of all-cause death (adjusted HR 1.50, 95% CI 1.00–2.25, *p* = 0.049) but similar risks of cardiovascular death and gastrointestinal bleeding when compared with DAPT.

**Conclusions:**

Among very elderly patients who were medically managed after AMI, single antiplatelet therapy had comparable protective effect as DAPT. But clopidogrel only strategy was associated with a higher risk of all-cause death.

**Electronic supplementary material:**

The online version of this article (10.1186/s12877-018-0777-4) contains supplementary material, which is available to authorized users.

## Background

Antiplatelet therapy has been considered the standard treatment for secondary prevention of ischemic events and mortality after acute myocardial infarction (AMI) [1, 2]. The benefit of dual antiplatelet therapy (DAPT) has been established in the Clopidogrel in Unstable Angina to Prevent Recurrent Events (CURE) trial, which showed improved outcomes among patients with non-ST elevation MI (NSTEMI) after treatment with DAPT [3]. Among patients with AMI, DAPT limits early steps of platelet aggregation and adhesion in the formation of coronary arteries thrombi, and improves clinical outcomes regardless of different reperfusion strategies [4].

In the era of percutaneous coronary intervention (PCI), the benefit of primary PCI or early invasive management do exist along with DAPT [5–7]. Nevertheless, a large proportion of patients with NSTEMI in clinical practice were managed medically [8, 9], with low rate of prescriptions of DAPT [10, 11]. Among the elderly AMI patients, conflicting results have emerged from different studies regarding to different PCI strategies, such as early aggressive versus initially conservative strategies [12–14]. In addition, the elderly AMI patients are frequently under-represented in clinical trials. Thus, there is uncertainty about efficacy and safety of DAPT in elderly AMI patients [15]. As a result, elderly population are less likely to receive evidence-based therapies [16], but are apt to be managed medically [17].

Less than one-half of medically managed patients with acute coronary syndrome (ACS) received clopidogrel on discharge [18, 19]. A relatively lower use of aspirin associated with increasing age was noted as well [20]. The impact of different anti-platelet strategies among medically managed elderly patient with AMI remains unclear. We therefore examined the effectiveness of DAPT and single antiplatelet therapy (aspirin only or clopidogrel only) in a real-world, community-based cohort of very elderly patients (above 85 years of age) who were medically managed after AMI.

## Methods

### Source of data

The National Health Insurance (NHI) program in Taiwan started in March 1995 and enrolled 22.60 million beneficiaries (more than 98% of inhabitants) at the end of 2007. NHI has reimbursed DAPT in all ACS patients including ST-segment elevation myocardial infarction (STEMI) and NSTEMI since October 1, 2007. Thus we included all patients who were admitted for AMI during the period through October 1, 2007 to December 31, 2010 from the NHI claims database. We extracted all of the records of the study subjects from the NHI claims database and linked to the National Death Registry for mortality outcomes using the identification number of each patient. To comply with data privacy regulations, personal identities were encrypted and all data were analyzed in a de-identified manner. The protocol for this study was approved by the Institutional Review Board of National Taiwan University Hospital.

### Study population

**Fig. 1:**
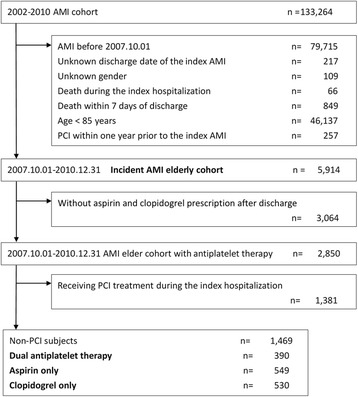
Patient flow diagram. Abbreviations: AMI, acute myocardial infarction; PCI, percutaneous coronary intervention

AMI was identified using International Classification of Disease- Clinical Modification, 9th version (ICD-9-CM) codes for STEMI (410.0~ 410.6, 410.8) and NSTEMI (410.7, 410.9). To identify true incident cases of AMI, patients had records of AMI within January 1, 2002 ~ September 30, 2007 were excluded. In addition, patients with unknown discharge date and gender were excluded. We also excluded patients who were younger than 85 years at index hospitalization for AMI, who died in hospital or within 7 days of discharge, and who had ever undergone PCI within 1 year prior to the index hospitalization. Patients with prescriptions of aspirin or clopidogrel after discharge were retained in the study cohort and were grouped into three groups including DAPT, aspirin only, and clopidogrel only, according to their first prescription at outpatient clinic after their index AMI hospitalization. The antiplatelet exposure duration should be 7 days or more and the date of the first prescription of antiplatelet agents was operationally defined as the index date. Those who did not have any prescription of anti-platelet agents within 28 days after discharge were excluded. A further 1381 patients were excluded due to receiving PCI during the index hospitalization. The remaining 1469 individuals comprised the final study cohort (Fig. 1).

### Potential confounders and outcomes

Potential confounders included age, sex, individual comorbidities such as diabetes mellitus, hypertension, congestive heart failure (CHF), peripheral artery disease (PAD), chronic pulmonary disease, renal disease, peptic ulcer disease and malignancy within the last 12 months prior to the index date in accordance with Charlson comorbidity measurements [21]. Use of statins, angiotensin-converting-enzyme inhibitors (ACEIs), angiotensin receptor blockers (ARBs), beta-blockers, diuretics and proton pump inhibitors (PPIs) within 60 days prior to the index date and at discharge of the index AMI hospitalization were also included as confounders. The clinical outcomes were all-cause death, cardiovascular death (ICD-9-CM codes 401–449 as the main cause of death) and gastrointestinal bleeding (ICD-9-CM codes 531.0, 531.2, 531.4, 531.6, 532.0, 532.2, 532.4, 532.6, 533.0, 533.2, 533.4, 533.6, 578.0, 578.1, 578.9). In Taiwan, the prescriptions of clopidogrel in ACS patients is reimbursed by NHI for only 9 months, rather than 12 months according to current international guidelines. [1, 2] Therefore, all the patients were followed until 9 months of treatment, occurrence of clinical outcomes, or December 31. 2010, whichever occurred first.

### Statistical analyses

For comparison of the baseline characteristics between the three groups, the 2-sample t-test was used for continuous variables and the chi-square test was employed for categorical variables. Pair-wise comparison approach using Cox proportional hazards regression model with adjustment for age, gender, AMI presentation, comorbidities and medications exposed was used to compare the relative risks of different clinical outcomes among the three groups. The test for proportional hazards assumption in Cox model was performed by Schoenfeld’s global test. If the proportional hazards assumption was violated, we would partition the relative risks as separate hazard ratios (HR's) within different follow-up time periods according to inspection of survival curves. DAPT, the standard treatment for AMI patients, was chosen a priori as the reference category. The survival curves of the three groups were illustrated by using the Kaplan-Meier method.

Even though professional medical associations in Taiwan have developed clinical guidelines concerning AMI management [22], variations in management of AMI among different levels of medical facilities could not be ignored. We utilized the shared frailty model to account for variability at hospital level under the assumption that patients clustered within the same medical facility shared the same level of frailty [23, 24]. Two shared frailty models were employed as sensitivity analyses. The first one controlled for 174 individual hospitals and the second one controlled for 11 different levels of hospitals where our study subjects were cared during their index AMI.

All the analyses were performed with SAS 9.4 software (SAS Institute Inc., Cary, North Carolina). A *p*-value<0.05 was considered statistically significant.

## Results

### Characteristics of study subjects

After application of selection criteria in the NHI claims database, we identified 1469 patients. Among them, 390 (27%) patients were prescribed with DAPT, 549 (37%) with aspirin only, and 530 (36%) with clopidogrel only (Fig. 1). The mean age was 88 years and the AMI presentations were comparable among three groups, with NSTEMI predominant but with less male patients in clopidogrel only group. Patients prescribed with aspirin only or clopidogrel only were more likely to have hypertension and chronic pulmonary disease but less likely to have diabetes and prescriptions of statins, ACEIs/ARBs, and beta-blockers at discharge in comparison with DAPT group. Clopidogrel only group were more likely to have history of peptic ulcer disease and had more prescriptions of proton pump inhibitors either at discharge or within the past 60 days prior to admission in comparison with DAPT group. Compared with DAPT group, aspirin only group were less likely to have prescriptions of statins within the past 60 days prior to admission (Table 1).

**Table 1:** Basic characteristics of study subjects

	DAPT	Aspirin only	*p* ^a^	Clopidogrel only	*p* ^b^
N	390	549		530	
Male (%)	49.2	50.6	0.64	42.8	0.026
Age, mean (yr)	88	88	0.31	89	0.54
Presentation					
NSTEMI (%)	85.9	83.8	0.07	88.1	0.12
STEMI (%)	14.1	16.2	0.11	11.9	0.13
Comorbidities					
Diabetes (%)	48.4	41.5	0.012	43.7	0.043
Hypertension (%)	27.4	40.1	< 0.001	41.3	< 0.001
CHF (%)	52.8	51.3	0.45	55.6	0.36
PAD (%)	5.9	8.5	0.15	6.1	0.17
Chronic pulmonary disease (%)	34.8	41.5	0.035	37.6	0.046
Renal disease	17.7	17.5	0.31	18.3	0.54
Peptic ulcer disease (%)	18.2	17.8	0.23	31.3	0.012
Malignancy (%)	7.7	9.2	0.24	11.1	0.053
Medications at discharge					
Statin (%)	37.9	20.6	< 0.001	24.9	< 0.001
ACEI/ARB (%)	73.8	59.2	< 0.001	61.5	< 0.001
Beta-blocker (%)	58.2	40.4	< 0.001	47.4	0.001
Diuretic (%)	69.5	63.9	0.08	71.9	0.43
Proton pump inhibitor (%)	11.8	15.7	0.09	38.5	< 0.001
Medications in past 60 days					
Statin (%)	12.8	7.3	0.005	9.81	0.15
ACEI/ARB (%)	38.7	37.3	0.67	37.7	0.76
Beta-blocker (%)	23.6	21.5	0.45	25.7	0.47
Diuretics (%)	25.1	31.0	0.05	29.4	0.15
Proton pump inhibitor (%)	6.2	5.7	0.74	10.2	0.030

^a^Comparison between aspirin only and DAPT groups

^b^Comparison between clopidogrel only and DAPT groups

*Abbreviations*, *ACEI* angiotensin-converting-enzyme inhibitor, *ARB* angiotensin receptor blocker, *CHF* congestive heart failure, *DATP* dual antiplatelet therapy, *NSTEMI* non-ST-elevation myocardial infarction, *PAD* peripheral artery disease, *STEMI* ST-elevation myocardial infarction

Additional file 1: eTable 1 also listed the distribution of study population in the 11 different levels of medical facilities among the 174 hospitals involved in this study.

### Clinical outcomes

Overall, the accumulated incidence was 13.8% for all-cause death, 7.9% for cardiovascular death and 4.2% for gastrointestinal bleeding (Table 2). Among three groups, patients treated with DAPT had numerically the lowest incidences of all-cause death (9.7%), cardiovascular death (6.1%), and gastrointestinal bleeding (3.1%) in comparison with aspirin only group (all-cause death, 12.9%; cardiovascular death, 8.7%; gastrointestinal bleeding, 4.9%) and clopidogrel only group (all-cause death, 17.9%; cardiovascular death, 8.5%; gastrointestinal bleeding, 4.3%). It is worth noting that the incidence of all-cause death in clopidogrel only group was nearly two-fold of that in DAPT group.

**Table 2:** Clinical outcomes associated with different antiplatelet therapies

	Total	DAPT	Aspirin only	Clopidogrel only
n	1469	390	549	530
All-cause death, n (%)	204 (13.8)	38 (9.7)	71 (12.9)	95 (17.9)
CV death, n (%)	117 (7.9)	24 (6.1)	48 (8.7)	45 (8.5)
GI bleeding, n (%)	62 (4.2)	12 (3.1)	27 (4.9)	23 (4.3)

*Abbreviations CV* cardiovascular, *DAPT* Dual antiplatelet therapy, *GI* gastrointestinal

**Fig. 2:**
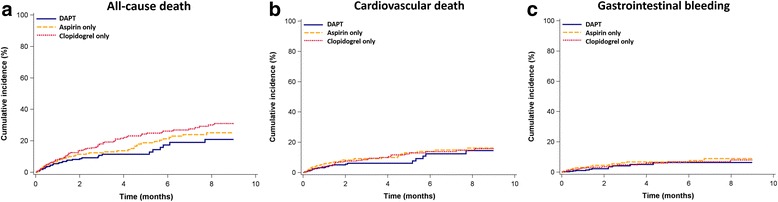
Kaplan–Meier curves for (**a**) all-cause death, (**b**) cardiovascular death, (**c**) gastrointestinal bleeding, among patients receiving different antiplatelet therapies. Abbreviations: DAPT, dual antiplatelet therapy

After adjusting for age, gender, AMI presentation, comorbidities and medications usage prior to and after index AMI, we found no difference in the risks of all-cause death (adjusted HR 1.20, 95% confidence interval [CI] 0.77–1.88, *p* = 0.42), cardiovascular death (adjusted HR 1.16, 95% CI 0.66–2.04, *p* = 0.60) and gastrointestinal bleeding (adjusted HR 1.66, 95% CI 0.77–3.61, *p* = 0.20) between aspirin only group and DAPT group. Nevertheless, patients treated with clopidogrel only possessed a higher risk of all-cause death (adjusted HR 1.50, 95% CI 1.00–2.25, *p* = 0.049) with comparable risks of cardiovascular death (adjusted HR 1.13, 95% CI 0.66–1.92, *p* = 0.65) and gastrointestinal bleeding (adjusted HR 0.96, 95% CI 0.44–2.09, *p* = 0.92) in comparison with patients treated with DAPT (Table 3). The Kaplan-Meier survival curves are illustrated in Fig. 2. The results remained unchanged in the two sensitivity analyses using the shared frailty model to control for characteristics of different levels of hospitals (Additional file 1: eTables 2 and 3).

**Table 3:** Relative risks of various clinical outcomes in patients receiving different antiplatelet therapies

	DAPT	Aspirin only				Clopidogrel only			
	HR	HR	95% CI	*P* ^a^	*P* for PH assumption	HR	95% CI	*P* ^b^	*P* for PH assumption
All-cause death	1	1.20	0.77–1.88	0.42	0.20	1.50	1.00–2.25	0.049	0.009
CV death	1	1.16	0.66–2.04	0.60	0.19	1.13	0.66–1.92	0.65	0.42
GI bleeding	1	1.66	0.77–3.61	0.20	0.16	0.96	0.44–2.09	0.92	0.48

^a^Comparison between aspirin only and DAPT groups

^b^Comparison between clopidogrel only and DAPT group

*Abbreviations*, CI confidence interval, *CV* cardiovascular, *DAPT* dual antiplatelet therapy, *GI* gastrointestinal, *HR* hazard ratio, *PH* proportional hazards

**Fig. 3:**
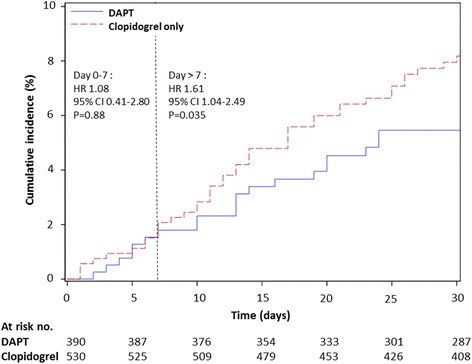
Kaplan–Meier curves for all-cause death among patients receiving dual antiplatelet therapy or clopidogrel only therapy. Abbreviations: CI, confidence interval; DAPT, dual antiplatelet therapy; HR, hazard ratio

Since the test for proportional hazards assumption was not satisfied concerning comparison of risk of all-cause death between clopidogrel only group and DAPT group (Table 3, *p* = 0.009), we chose the 7th day after the index date as cut-off time point for partition of separate HR’s based on inspection of the survival curves (Figs. 2 and 3). Of note, during the first 7 days, use of clopidogrel only was not associated with an increased risk of all-cause death in comparison with DAPT group (adjusted HR 1.08, 95% CI 0.41–2.80, *p* = 0.88). However, the risk of all-cause death in clopidogrel only group became statistically significant after the 7th day post-index date as compared with DAPT (adjusted HR 1.61, 95% CI 1.04–2.49, *p* = 0.035) (Fig. 3).

Our results and abstract were submitted to the 21st Cardiovascular Summit Transcatheter Cardiovascular Therapeutics Asia Pacific (TCTAP) and published in the supplement of Journal of the American College of Cardiology [25].

## Discussion

Currently, DAPT represents the mainstay of treatment strategy in post-AMI survivors [26, 27]. However, the efficacy of DAPT in elderly population remains uncertain because elderly patients are underrepresented in randomized trials for cardiovascular disease and age may alter the balance of risk and benefit of therapeutic strategies [28]. Furthermore, physicians are challenged with peculiar pharmacokinetic and pharmacodynamic mechanisms of altered drug response in the late age of life. Overall, the elderly is generally more vulnerable to the adverse effects of anti-thrombotic drugs. The aim of this study was to investigate different clinical outcomes among very elderly AMI survivors who were older than 85 years and managed medically with DAPT, aspirin only or clopidogrel only. Our results indicated that these three anti-thrombotic strategies had similar risks of cardiovascular death and gastrointestinal bleeding. Nevertheless, clopidogrel only strategy was associated with a higher risk of all-cause death when compared with DAPT strategy especially after 7 days of usage.

Physicians may prefer medical and more conservative treatment for elderly AMI patients because of varieties of electrocardiographic abnormalities [29], cognitive impairment or social constraints [30], and coexisting comorbid geriatric conditions. Our study clearly showed that patients in DAPT group were less likely to have comorbidities such as hypertension, chronic pulmonary disease, and peptic ulcer disease, but received more aggressive treatments including statins, ACEIs/ARBs, and beta-blockers. It was not surprising that patients in DAPT group had the lowest incidences of all the three clinical outcomes compared with the other two groups in crude results. While adjusting for confounders, our analysis demonstrated no difference in the risks of all-cause death and cardiovascular death between DAPT and aspirin only users. In the CURE trial, those older than 65 years of age still showed significant absolute and relative risk reduction (2.0% and 13.1%, respectively) of the primary end point with the addition of clopidogrel on aspirin therapy [3]. The loss of protective effect of DAPT strategy compared with aspirin only or clopidogrel only strategies in our study might be owing to the very elderly (> 85 years) nature in our study population. Further investigations will be needed to provide conclusive results regarding a possible effect modification by age factor on the clinical impact of DAPT.

In the Dual Antiplatelet Therapy Study, the author noted higher HR of all-cause death among patients receiving thienopyridine compared with placebo group (HR = 2.23, *p* = 0.002) [31]. More cancer-related deaths among patients treated with thienopyridine was considered to contribute the higher risk of all-cause death. One study comparing long-term thienopyridine therapy with placebo in patients with lacunar stroke also identified an unexpected increase in mortality [32]. Our patients were the oldest and those receiving clopidogrel only strategy seemed to have more prevalent malignancy with a trend toward statistical significance (11.1% vs. 7.7%, *p* = 0.053, in comparison with DAPT group). Although we had included malignancy as one of the confounders during regression analysis, unmeasured factors still might exist. Consequently, the imbalance in the background of our cohort populations may probably explain the increased all-cause mortality associated with clopidogrel only strategy. In addition, we observed different risk ratios concerning all-cause death before and after 7 days of treatment when comparing clopidogrel only with DAPT groups. One possible explanation was that the beneficial effect of antiplatelet therapy conferred by clopidogrel diminished gradually when the acute inflammatory phase associated with AMI dissipated after discharge.

In term of the risk of gastrointestinal bleeding, there was no significant difference between single antiplatelet treatments and DAPT in our study. DAPT has been shown to increase the risk of gastrointestinal bleeding in many trials [3, 33] while PPI therapy has been shown to decrease this risk [34]. Furthermore, the guideline recommended gastroprotection with PPIs for all patients receiving DAPT [35]. Among AMI survivors in our study, a part of patients had been prescribed with PPIs which might contribute to no difference in the risk of gastrointestinal bleeding between three different antiplatelet strategies.

Frail and vulnerable elderly patients, along with comorbidities and disabilities, are easily neglected by studies and trials. In the respect of use of antiplatelet, there was no similar research in Asian population. Given the culture influence and preferred conservative treatment, our study reflected the real-world practice and provided evidence for various antiplatelet strategies in these specific elderly AMI patients without PCI.

### Limitations

This study also has important limitations that should be acknowledged. Firstly, our results were limited by its non-randomized comparisons, selection bias and an uneven distribution of risk factors. Although we performed regression adjustment with all the covariates listed in Table 1, the results might be still confounded by other underlying conditions which were not included in analysis. Secondly, we estimated HR's of three different outcomes with six pairwise comparisons, which raised the question of statistical significance in multiple testing. While the most common way to control the type I error is using the Bonferroni correction to reduce the risk of false significance, *p*-value adjustments by the Bonferroni correction is also considered to be overly conservative and leads to a very high rate of false negatives [36, 37]. In addition, the eligible subjects in our analysis was quite small though there was a large number of patients in our database. It might result in wide confidence intervals of estimates. Therefore, we provided conventional statistical inference instead of adjusting the *p*-value to point out an interesting observation which needed further validation. In other words, the aim of our study was to generate a hypothesis or idea to inspire further clinical research. Thirdly, the NHI claims database does not include self-purchased drugs over the counter. Therefore, the possibility of misclassification could not be excluded particularly for self-purchases of aspirin over the counter in clopidogrel only group. However, any resulting misclassification was likely to result in a dilution of the true effect. Accordingly, our conclusion that clopidogrel users had a statistically significant higher risk of all-cause death compared with DAPT users seemed robust under this presumption. Fourthly, guidelines also recommend the use of prasugrel and ticagrelor combined with aspirin in ACS patients. Because prasugrel is not approved for use in Taiwan and ticagrelor is reimbursed by Taiwan NHI since 2013, these two medications were not examined in our study. Finally, the incidence of intracranial hemorrhage was too small to be included in our analysis.

## Conclusion

Among very elderly AMI patients not receiving revascularization therapy, single antiplatelet therapy, either aspirin only or clopidogrel only, possessed a comparable protective effect on cardiovascular death and a similar risk of gastrointestinal bleeding while comparing with clopidogrel-plus-aspirin DAPT. However, clopidogrel only strategy was associated with an increase in the risk of all-cause death especially after 7 days of treatment.

## Additional file

Additional file 1: **eTable 1.** The care facilities of study subjects during the index acute myocardial infarction. **eTable 2.** Relative risks of various clinical outcomes in patients receiving different antiplatelet therapies using shared frailty model controlling for 174 individual hospitals. **eTable 3.** Relative risks of various clinical outcomes in patients receiving different antiplatelet therapies using shared frailty model controlling for 11 different levels of hospitals. (DOCX 20 kb)

## Abbreviations

ACEI: Angiotensin-converting-enzyme inhibitor
ACS: Acute coronary syndrome
AMI: Acute myocardial infarction
ARB: Angiotensin receptor blocker
CHF: Congestive heart failure
CI: Confidence interval
CURE: The Clopidogrel in Unstable Angina to Prevent Recurrent Events trial
DAPT: Dual antiplatelet therapy
HR: Hazard ratio
ICD-9-CM: International Classification of Disease- Clinical Modification, 9th version
NHI: National Health Insurance
NSTEMI: Non-ST segment elevation myocardial infarction
PAD: Peripheral artery disease
PCI: Percutaneous coronary intervention
PPI: Proton pump inhibitor
STEMI: ST-segment elevation myocardial infarction

## References

[1] Roffi M, Patrono C, Collet JP, Mueller C, Valgimigli M, Andreotti F, Bax JJ, Borger MA, Brotons C, Chew DP (2016). ESC guidelines for the management of acute coronary syndromes in patients presenting without persistent ST-segment elevation: task force for the Management of Acute Coronary Syndromes in patients presenting without persistent ST-segment elevation of the European Society of Cardiology (ESC). Eur Heart J.

[2] Ibanez B, James S, Agewall S, Antunes MJ, Bucciarelli-Ducci C, Bueno H, Caforio ALP, Crea F, Goudevenos JA, Halvorsen S (2018). 2017 ESC guidelines for the management of acute myocardial infarction in patients presenting with ST-segment elevation: the task force for the management of acute myocardial infarction in patients presenting with ST-segment elevation of the European Society of Cardiology (ESC). Eur Heart J.

[3] Yusuf S, Zhao F, Mehta SR, Chrolavicius S, Tognoni G, Fox KK (2001). Effects of clopidogrel in addition to aspirin in patients with acute coronary syndromes without ST-segment elevation. N Engl J Med.

[4] Sabatine MS, Cannon CP, Gibson CM, Lopez-Sendon JL, Montalescot G, Theroux P, Claeys MJ, Cools F, Hill KA, Skene AM (2005). Addition of clopidogrel to aspirin and fibrinolytic therapy for myocardial infarction with ST-segment elevation. N Engl J Med.

[5] Mehta SR, Yusuf S, Peters RJ, Bertrand ME, Lewis BS, Natarajan MK, Malmberg K, Rupprecht H, Zhao F, Chrolavicius S (2001). Effects of pretreatment with clopidogrel and aspirin followed by long-term therapy in patients undergoing percutaneous coronary intervention: the PCI-CURE study. Lancet.

[6] von Beckerath N, Taubert D, Pogatsa-Murray G, Schomig E, Kastrati A, Schomig A (2005). Absorption, metabolization, and antiplatelet effects of 300-, 600-, and 900-mg loading doses of clopidogrel: results of the ISAR-CHOICE (intracoronary stenting and antithrombotic regimen: choose between 3 high oral doses for immediate Clopidogrel effect) trial. Circulation.

[7] Di Sciascio G, Patti G, Pasceri V, Gatto L, Colonna G, Montinaro A (2010). Effectiveness of in-laboratory high-dose clopidogrel loading versus routine pre-load in patients undergoing percutaneous coronary intervention: results of the ARMYDA-5 PRELOAD (antiplatelet therapy for reduction of MYocardial damage during angioplasty) randomized trial. J Am Coll Cardiol.

[8] de Winter RJ, Windhausen F, Cornel JH, Dunselman PH, Janus CL, Bendermacher PE, Michels HR, Sanders GT, Tijssen JG, Verheugt FW (2005). Early invasive versus selectively invasive management for acute coronary syndromes. N Engl J Med.

[9] Maddox TM, Ho PM, Tsai TT, Wang TY, Li S, Peng SA, Wiviott SD, Masoudi FA, Rumsfeld JS (2012). Clopidogrel use and hospital quality in medically managed patients with non-ST-segment-elevation myocardial infarction. Circ Cardiovasc Qual Outcomes.

[10] Banihashemi B, Goodman SG, Yan RT, Welsh RC, Mehta SR, Montalescot G, Kornder JM, Wong GC, Gyenes G, Steg PG (2009). Underutilization of clopidogrel and glycoprotein IIb/IIIa inhibitors in non-ST-elevation acute coronary syndrome patients: the Canadian global registry of acute coronary events (GRACE) experience. Am Heart J.

[11] Rao RV, Goodman SG, Yan RT, Spencer FA, Fox KA, DeYoung JP, Rose B, Grondin FR, Gallo R, Gore JM (2009). Temporal trends and patterns of early clopidogrel use across the spectrum of acute coronary syndromes. Am Heart J.

[12] Bach RG, Cannon CP, Weintraub WS, DiBattiste PM, Demopoulos LA, Anderson HV, DeLucca PT, Mahoney EM, Murphy SA, Braunwald E (2004). The effect of routine, early invasive management on outcome for elderly patients with non-ST-segment elevation acute coronary syndromes. Ann Intern Med.

[13] Savonitto S, Cavallini C, Petronio AS, Murena E, Antonicelli R, Sacco A, Steffenino G, Bonechi F, Mossuti E, Manari A (2012). Early aggressive versus initially conservative treatment in elderly patients with non-ST-segment elevation acute coronary syndrome: a randomized controlled trial. JACC Cardiovasc Interv.

[14] Tegn N, Abdelnoor M, Aaberge L, Endresen K, Smith P, Aakhus S, Gjertsen E, Dahl-Hofseth O, Ranhoff AH, Gullestad L (2016). Invasive versus conservative strategy in patients aged 80 years or older with non-ST-elevation myocardial infarction or unstable angina pectoris (after eighty study): an open-label randomised controlled trial. Lancet.

[15] Lee PY, Alexander KP, Hammill BG, Pasquali SK, Peterson ED (2001). Representation of elderly persons and women in published randomized trials of acute coronary syndromes. JAMA.

[16] Zaman MJ, Stirling S, Shepstone L, Ryding A, Flather M, Bachmann M, Myint PK (2014). The association between older age and receipt of care and outcomes in patients with acute coronary syndromes: a cohort study of the myocardial Ischaemia National Audit Project (MINAP). Eur Heart J.

[17] De Servi S, Cavallini C, Dellavalle A, Santoro GM, Bonizzoni E, Marzocchi A, Politi A, Pesaresi A, Mariani M, Chierchia S (2004). Non-ST-elevation acute coronary syndrome in the elderly: treatment strategies and 30-day outcome. Am Heart J.

[18] Amsterdam EA, Peterson ED, Ou FS, Newby LK, Pollack CV, Gibler WB, Ohman EM, Roe MT (2009). Comparative trends in guidelines adherence among patients with non-ST-segment elevation acute coronary syndromes treated with invasive versus conservative management strategies: results from the CRUSADE quality improvement initiative. Am Heart J.

[19] Mehta RH, Roe MT, Chen AY, Lytle BL, Pollack CV, Brindis RG, Smith SC, Harrington RA, Fintel D, Fraulo ES (2006). Recent trends in the care of patients with non-ST-segment elevation acute coronary syndromes: insights from the CRUSADE initiative. Arch Intern Med.

[20] Medina HM, Cannon CP, Zhao X, Hernandez AF, Bhatt DL, Peterson ED, Liang L, Fonarow GC (2011). Quality of acute myocardial infarction care and outcomes in 33,997 patients aged 80 years or older: findings from get with the guidelines-coronary artery disease (GWTG-CAD). Am Heart J.

[21] Quan H, Sundararajan V, Halfon P, Fong A, Burnand B, Luthi JC, Saunders LD, Beck CA, Feasby TE, Ghali WA (2005). Coding algorithms for defining comorbidities in ICD-9-CM and ICD-10 administrative data. Med Care.

[22] Yi-Heng Li H-IY, Tsai C-T, Liu P-Y, Lin T-H, Wu T-C, Hung K-C, Hsieh Y-C, Mar G-Y, Fang C-Y, Chiu K-M, Cheng J-J, Che J-H (2012). Guidelines of the Taiwan Society of Cardiology (TSOC) for the management of ST-segment elevation myocardial infarction. Acta Cardiologica Sinica.

[23] Vaupel JW, Manton KG, Stallard E (1979). The impact of heterogeneity in individual frailty on the dynamics of mortality. Demography.

[24] Zeng D, Chen Q, Ibrahim JG (2009). Gamma frailty transformation models for multivariate survival times. Biometrika.

[25] Ting-Tse L, Liao M-T, Lai C-L (2016). Comparable effectiveness between aspirin, Clopidogrel and dual antiplatelet therapy in very elderly patients with medically managed acute myocardial infarction. J Am Coll Cardiol.

[26] Steg PG, James SK, Atar D, Badano LP, Blomstrom-Lundqvist C, Borger MA, Di Mario C, Dickstein K, Ducrocq G, Fernandez-Aviles F (2012). ESC guidelines for the management of acute myocardial infarction in patients presenting with ST-segment elevation. Eur Heart J.

[27] Roffi M, Patrono C, Collet JP, Mueller C, Valgimigli M, Andreotti F, Bax JJ, Borger MA, Brotons C, Chew DP (2016). 2015 ESC guidelines for the management of acute coronary syndromes in patients presenting without persistent ST-segment elevation: task force for the Management of Acute Coronary Syndromes in patients presenting without persistent ST-segment elevation of the European Society of Cardiology (ESC). Eur Heart J.

[28] Roe MT, Goodman SG, Ohman EM, Stevens SR, Hochman JS, Gottlieb S, Martinez F, Dalby AJ, Boden WE, White HD (2013). Elderly patients with acute coronary syndromes managed without revascularization: insights into the safety of long-term dual antiplatelet therapy with reduced-dose prasugrel versus standard-dose clopidogrel. Circulation.

[29] Rogers WJ, Bowlby LJ, Chandra NC, French WJ, Gore JM, Lambrew CT, Rubison RM, Tiefenbrunn AJ, Weaver WD (1994). Treatment of myocardial infarction in the United States (1990 to 1993). Observations from the National Registry of myocardial infarction. Circulation.

[30] Gurwitz JH, McLaughlin TJ, Willison DJ, Guadagnoli E, Hauptman PJ, Gao X, Soumerai SB (1997). Delayed hospital presentation in patients who have had acute myocardial infarction. Ann Intern Med.

[31] Mauri L, Kereiakes DJ, Yeh RW, Driscoll-Shempp P, Cutlip DE, Steg PG, Normand SL, Braunwald E, Wiviott SD, Cohen DJ (2014). Twelve or 30 months of dual antiplatelet therapy after drug-eluting stents. N Engl J Med.

[32] Benavente OR, Hart RG, McClure LA, Szychowski JM, Coffey CS, Pearce LA (2012). Effects of clopidogrel added to aspirin in patients with recent lacunar stroke. N Engl J Med.

[33] Connolly SJ, Pogue J, Hart RG, Hohnloser SH, Pfeffer M, Chrolavicius S, Yusuf S (2009). Effect of clopidogrel added to aspirin in patients with atrial fibrillation. N Engl J Med.

[34] Lai KC, Lam SK, Chu KM, Wong BC, Hui WM, Hu WH, Lau GK, Wong WM, Yuen MF, Chan AO (2002). Lansoprazole for the prevention of recurrences of ulcer complications from long-term low-dose aspirin use. N Engl J Med.

[35] Abraham NS, Hlatky MA, Antman EM, Bhatt DL, Bjorkman DJ, Clark CB, Furberg CD, Johnson DA, Kahi CJ, Laine L (2010). ACCF/ACG/AHA 2010 expert consensus document on the concomitant use of proton pump inhibitors and thienopyridines: a focused update of the ACCF/ACG/AHA 2008 expert consensus document on reducing the gastrointestinal risks of antiplatelet therapy and NSAID use. A report of the American College of Cardiology Foundation task force on expert consensus documents. J Am Coll Cardiol.

[36] Feise RJ (2002). Do multiple outcome measures require p-value adjustment?. BMC Med Res Methodol.

[37] Bland JM, Altman DG (1995). Multiple significance tests: the Bonferroni method. BMJ.

