# Supplementary material for: Single and dual antiplatelet therapy in elderly patients of medically managed myocardial infarction
Source: BMC Geriatr. 2018 Apr 5;18:86. doi: 10.1186/s12877-018-0777-4 (PMC5887242; doi:10.1186/s12877-018-0777-4)
Supplement: Supplementary file 1 — eTable 1. The care facilities of study subjects during the index acute myocardial infarction. eTable 2. Relative risks of various clinical outcomes in patients receiving different antiplatelet therapies using shared frailty model controlling for 174 individual hospitals. eTable 3. Relative risks of various clinical outcomes in patients receiving different antiplatelet therapies using shared frailty model controlling for 11 different levels of hospitals. (DOCX 20 kb) [file 12877_2018_777_MOESM1_ESM.docx]

**Supplementary Materials**

**eTable 1.** The care facilities of study subjects during the index acute myocardial infarction.

**Shared Frailty Model Analysis**

**eTable 2.** Relative risks of various clinical outcomes in patients receiving different antiplatelet therapies using shared frailty model controlling for 174 individual hospitals.

**eTable 3.** Relative risks of various clinical outcomes in patients receiving different antiplatelet therapies using shared frailty model controlling for 11 different levels of hospitals.

**eTable 1.** The care facilities of study subjects during the index acute myocardial infarction.

| Care Facilities* | DAPT  N=390 | Aspirin only  N=549 | Clopidogrel only  N=530 |
| --- | --- | --- | --- |
| Hospitals subordinate to the Ministry of Health and Welfare (%) | 10.5 | 14.8 | 9.3 |
| County & City Hospitals (%) | 1.3 | 1.3 | 0.6 |
| Hospitals Affiliated with Public Medical Schools (%) | 9.2 | 3.5 | 6.6 |
| Civilian Clinics of Military Hospitals (%) | 4.1 | 5.8 | 3.6 |
| Veterans Hospitals (%) | 12.1 | 10.9 | 6.8 |
| Hospitals Affiliated with Enterprises (%) | 4.4 | 6.6 | 3.4 |
| Non-Profit Proprietary Hospitals (%) | 33.3 | 30.2 | 43.8 |
| Hospitals Affiliated with Non-Profit Proprietary Religious Organizations (%) | 2.1 | 3.5 | 2.3 |
| Hospitals Affiliated with Private Medical Schools (%) | 12.1 | 6.0 | 12.1 |
| Hospitals Affiliated with Other Non-Profit Proprietary Organizations (%) | 2.8 | 2.6 | 1.1 |
| Private Hospitals (%) | 8.2 | 14.9 | 10.6 |

*Total hospital number: 174.

Abbreviation: DAPT, dual antiplatelet therapy.

**eTable 2.** Relative risks of various clinical outcomes in patients receiving different antiplatelet therapies using shared frailty model controlling for 174 individual hospitals.

|  | DAPT |  | Aspirin only | | | |  | | Clopidogrel only | | | | | |  |
| --- | --- | --- | --- | --- | --- | --- | --- | --- | --- | --- | --- | --- | --- | --- | --- |
| Drugs | HR |  | HR | 95% CI | | P* | |  | | HR | | 95% CI | | p† | |
| All-cause death | 1 |  | 1.21 | 0.77-1.89 | 0.41 | |  | | 1.51 | | 1.01-2.27 | | 0.046 | |  |
| CV death | 1 |  | 1.16 | 0.66-2.04 | 0.61 | |  | | 1.12 | | 0.66-1.91 | | 0.67 | |  |
| GI bleeding | 1 |  | 1.68 | 0.78-3.63 | 0.19 | |  | | 0.95 | | 0.44-2.08 | | 0.90 | |  |

*Comparison between aspirin only and DAPT groups.

†Comparison between clopidogrel only and DAPT groups.

Abbreviations: CI, confidence interval; CV, cardiovascular; DAPT, dual antiplatelet therapy; GI, gastrointestinal; HR, hazard ratio.

**eTable 3.** Relative risks of various clinical outcomes in patients receiving different antiplatelet therapies using shared frailty model controlling for 11 different levels of hospitals.

|  | DAPT |  | Aspirin only | | | |  | | Clopidogrel only | | | | | |  |
| --- | --- | --- | --- | --- | --- | --- | --- | --- | --- | --- | --- | --- | --- | --- | --- |
| Drugs | HR |  | HR | 95% CI | | P* | |  | | HR | | 95% CI | | p† | |
| All-cause death | 1 |  | 1.21 | 0.77-1.89 | 0.41 | |  | | 1.51 | | 1.01-2.27 | | 0.046 | |  |
| CV death | 1 |  | 1.16 | 0.66-2.04 | 0.60 | |  | | 1.13 | | 0.67-1.92 | | 0.65 | |  |
| GI bleeding | 1 |  | 1.66 | 0.77-3.57 | 0.20 | |  | | 0.95 | | 0.44-2.07 | | 0.90 | |  |

*Comparison between aspirin only and DAPT groups.

†Comparison between clopidogrel only and DAPT groups.

Abbreviations: CI, confidence interval; CV, cardiovascular; DAPT, dual antiplatelet therapy; GI, gastrointestinal; HR, hazard ratio.
